# Left bundle branch pacing as an alternative to biventricular pacing for cardiac resynchronisation therapy

**DOI:** 10.1007/s12471-022-01712-9

**Published:** 2022-08-03

**Authors:** L. M. Rademakers, J. L. P. M. van den Broek, F. A. Bracke

**Affiliations:** Department of Cardiology, Catharina Hospital, Eindhoven, The Netherlands

**Keywords:** Left bundle branch pacing, Physiological pacing, Cardiac resynchronisation therapy, Biventricular pacing

## Abstract

**Background:**

Left bundle branch pacing (LBBP) is a novel physiological pacing technique which may serve as an alternative to biventricular pacing (BVP) for the delivery of cardiac resynchronisation therapy (CRT). This study assessed the feasibility and outcomes of LBBP in comparison to BVP.

**Methods:**

LBBP was attempted in 40 consecutive patients as the first-line method for delivering CRT. To evaluate LBBP versus BVP, 40 patients with identical inclusion criteria who received BVP were compared with the LBBP group. Acute success rate, complications, functional and echocardiographic outcomes as well as hospitalisation for heart failure and all-cause mortality 6 months after implantation were evaluated.

**Results:**

LBBP was successfully performed in 31 (78%) patients and resulted in significant QRS narrowing (from 166 ± 16 to 123 ± 18 ms, *p* < 0.001), improvement in left ventricular ejection fraction (LVEF; from 28 ± 8 to 43 ± 12%, *p* < 0.001) and New York Heart Association functional class (from 2.8 ± 0.5 to 1.6 ± 0.6, *p* < 0.001) at 6 months. No LBBP-related complications occurred. Compared to BVP, LBBP resulted in a greater reduction in QRS duration (44 ± 17 vs 15 ± 26 ms, *p* < 0.001) with comparable absolute improvement in LVEF (15.2 ± 11.7 vs 9.6 ± 12.1%, *p* = 0.088). Hospitalisation for heart failure and all-cause mortality were similar in the two groups.

**Conclusions:**

LBBP is feasible and was safe in 78% of patients with favourable electrical resynchronisation and functional improvement and may serve as an alternative to BVP.

**Supplementary Information:**

The online version of this article (10.1007/s12471-022-01712-9) contains supplementary material, which is available to authorized users.

## What’s new?

- Left bundle branch pacing (LBBP) is a novel physiological pacing technique which may serve as an alternative to biventricular pacing for the delivery of cardiac resynchronisation therapy (CRT).
- LBBP was shown to be a safe and feasible method of delivering CRT in 78% of patients with favourable electrical resynchronisation and functional improvement.

## Introduction

Physiological pacing is characterised by direct stimulation of the intrinsic His-Purkinje system and results in physiological ventricular depolarisation and repolarisation. In 2017, Huang et al. [1] first demonstrated that, by pacing beyond the region of block, left bundle branch pacing (LBBP) could achieve complete correction of left bundle branch block (LBBB) and improved left ventricular (LV) function in a patient with heart failure and LBBB. Therefore, this technique may serve as an alternative to biventricular pacing (BVP) [2–4]. The present study aimed to assess the implant, electrocardiogram (ECG) and pacing parameters, as well as the echocardiographic and clinical response to LBBP-delivered cardiac resynchronisation therapy (CRT) in patients with LBBB and symptomatic heart failure. The findings were compared to those of BVP.

## Methods

### Patient selection

From January 2020 to September 2020, 40 consecutive patients who were in sinus rhythm with New York Heart Association (NYHA) class II–IV heart failure, reduced left ventricular ejection fraction (LVEF; ≤ 35%) and complete LBBB according to the Strauss criteria [5] with QRS > 140 ms in men and > 130 ms in women underwent attempted LBBP as the first-line method for delivering CRT. These patients were prospectively studied. Prior to the implantation procedure the operators discussed the non-standard but potentially more physiological nature of conduction system pacing with the patients, and all patients participating in this study provided informed consent. Patients who refused LBBP received conventional BVP and were excluded from this study. The study protocol was approved by both our institutional ethics committee and an independent medical research ethics committee (MEC-U) with an in-hospital independent monitoring committee. If LBBP was not satisfactory, BVP was performed as a bail-out procedure. In order to evaluate LBBP versus BVP, 40 consecutive patients with identical inclusion criteria who received BVP from January 2019 to September 2019 were retrospectively analysed and compared with the LBBP group. Patients were required to be in sinus rhythm in order to be included in the study but were allowed to have a history of paroxysmal or persistent atrial fibrillation. Permanent atrial fibrillation was an exclusion criterion. Patients were classified as having an ischaemic cardiomyopathy if they had had a previous myocardial infarction or revascularisation. All patients were on maximally tolerated heart failure medication for at least 3 months prior to device implantation.

### Procedure

All device implantations were performed with the patient under local anaesthesia after perioperative administration of 2 g intravenous cefazoline. If patients were on direct oral anticoagulant therapy, treatment was interrupted 24 h before implantation. Vitamin K antagonists were generally not interrupted, and device implantation was performed if the international normalised ratio did not exceed 3.0. Cephalic vein access for all leads using a modified Seldinger technique was the standard approach [6]. Alternative access routes (i.e. axillary or subclavian vein puncture) were reserved for bail-out procedures. The procedures were performed by two operators (L.M.R and F.A.B) with experience in BVP and LBBP.

### Biventricular lead implantation

After cannulation of the coronary sinus and venography, selection of the quadripolar LV lead was based on the decision of the implanting physician. Bipolar leads were not used. The LV lead was placed preferably in a basal position in a lateral or posterolateral vein, whereas the right ventricular (RV) lead was positioned in the RV septum or apex. Devices were programmed in DDD mode with an atrioventricular (AV) delay optimised for the shortest paced QRS duration.

### LBB lead implantation

**Fig. 1:**
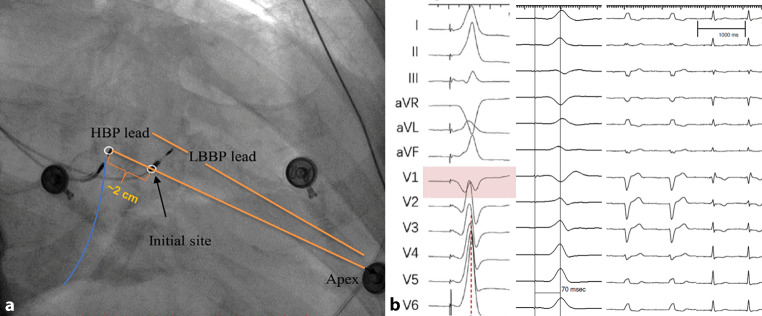
**a**, **b** Finding the optimal site for proximal left bundle branch pacing (*LBBP*) and its electrocardiographic characteristics. **a** Location of the His-bundle pacing (*HBP*) lead and LBBP lead in the right anterior oblique 30° view. *Blue line* indicates location of tricuspid valve and *orange lines* indicate demarcation of interventricular septum. **b** *Left panel* Unipolar pace mapping at the interventricular septum before lead fixation, demonstrating ‘W’ pattern with a mid-notch in the QRS complex in lead V1; *middle panel* measurement of left ventricular activation time during bipolar pacing at a 200 mm/s sweep speed; *right panel* 12-lead ECG with baseline LBB block (2 beats) followed by LBBP (2 beats) at a 25 mm/s sweep speed. Part of figure modified after Huang et al. [7] and Zhang et al. [25]

LBBP was performed using the SelectSecure Model 3830, 74-cm pacing lead (Medtronic, Minneapolis, MN, USA) and the C315HIS delivery sheath (Medtronic). Our implantation method was based on the descriptions of Huang et al. [7]. After positioning the C315HIS sheath with the pacing lead in the right ventricle, unipolar pace mapping was used to find the site for pacing lead implantation, i.e. (1) the presence of aVR/aVL discordance with the R wave in lead II more positive than in lead III, or the R wave positive in lead II and negative in lead III, preferably in combination with (2) a paced ECG QRS morphology in lead V1 showing a ‘W’ pattern with a mid-notch (see Fig. 1a, b). Next, using a left anterior oblique 30° fluoroscopic projection the sheath was positioned perpendicular to the interventricular septum and the pacing lead was fixated in the septum under intermittent fluoroscopic guidance. Unipolar pacing was performed to assess the paced QRS morphology and pacing impedance until the paced QRS morphology resembled a right bundle branch block (RBBB) or RBB conduction delay pattern in V1 (QR pattern). The left ventricular activation time (LVAT, time interval from unipolar pacing spike to R‑wave peak in lead V5 or V6) at different outputs (usually 1.5 V and 5.0 V) was tested and recorded (200 mm/s sweep speed) on an electrophysiology recording system (Prucka Cardiolab, GE Healthcare, Waukesha, WI, USA). LBBP was confirmed when the paced QRS morphology demonstrated both an RBBB morphology (QR or rSR’) and an LVAT that shortened abruptly with increasing output (or remained shortest and constant at both low and high outputs). Although there was no validated cut-off, we regarded LVAT ≤ 90 ms to be an indicator of LBB capture [8]. LBBP was considered unsuccessful if the above-mentioned criteria of QRS morphology and LVAT cut-off could not be met.

At the physician’s discretion an additional RV back-up lead could be placed in the septum or apex. In CRT devices the LBB lead was connected to the LV port and the RV (shock) lead to the RV port. In dual-chamber pacemakers, the atrial lead was connected to the atrial port and the LBB lead to the ventricular port. In dual-chamber implantable cardioverter-defibrillators (ICDs), the LBB lead was connected to the pace/sense port of the device and the pace/sense part of the IS-1/DF‑1 shock lead was capped. Devices were programmed in DDD mode with the AV delay optimised for the shortest paced QRS duration. LV to RV offsets were programmed to achieve functional RV non-capture.

### Follow-up data

All patients underwent follow-up at 1, 3 and 6 months at the outpatient device clinic. NYHA functional class was assessed preimplantation and at 6 months. Clinical response to CRT was defined as an improvement in NYHA class by at least 1 class without hospital admission for heart failure. Echocardiography was performed during preoperative and postoperative follow-up for analysis of LV dimensions and LVEF. The latter was calculated using the biplane Simpson’s method [9]. All echocardiograms were anonymised and randomly analysed by one operator (L.M.R.), who was blinded to the treatment arm after the last patient had completed follow-up. Echocardiographic response was defined as an at least 5% increase in LVEF between baseline and follow-up echocardiogram at 6 months. Baseline and paced 12-lead ECGs were recorded at each follow-up. Device interrogations were performed for analysis of R‑wave amplitudes, capture thresholds, lead impedances and percentages of LBBP or BVP. Hospitalisation for heart failure and all-cause mortality were monitored during follow-up.

### Endpoints

The main endpoints of this study were the acute LBBP success rate, safety and clinical and echocardiographic response to LBBP-delivered CRT after 6 months. Secondary endpoints included hospitalisation for heart failure, all-cause mortality and comparison to the BVP cohort.

### Statistics

Continuous data are presented as mean and standard deviation and discrete variables as counts and percentages, unless otherwise stated. Continuous data were compared using a Student’s *t*-test or repeated-measures analysis of variance. Comparisons of continuous variables within groups were carried out using the paired Student’s *t*-test or Wilcoxon signed-rank test. Discrete variables were analysed with the Fisher’s exact test. A two-sided *p*-value < 0.05 was considered statistically significant. No missing data imputation was performed. Analyses were performed using SPSS Statistics (v.25, IBM Corp., Armonk, NY, USA).

## Results

Baseline characteristics are summarised in Tab. 1. A total of 40 consecutive patients underwent attempted LBBP. Their mean age was 68 years (48% male) and the mean LVEF was 28 ± 8%, with 28% of patients having an ischaemic cardiomyopathy. Two-thirds of the patients were in NYHA class III heart failure. The mean baseline QRS duration was 166 ± 16 ms.

**Table 1:** Baseline characteristics

Parameter	LBBP (*n* = 40)	BVP (*n* = 40)	*p‑value*
*Age, years*	68 ± 13	71 ± 9	0.225
*Sex*			0.301
– Male	19 (48%)	27 (68%)	
*– *Female	21 (52%)	13 (32%)	
*Medical history*			0.869
– Hypertension	34 (85%)	32 (80%)	
– Chronic kidney disease	4 (10%)	6 (15%)	
– Diabetes mellitus	8 (20%)	9 (23%)	
– Coronary artery disease	18 (45%)	20 (50%)	
– Atrial fibrillation/flutter	9 (23%)	13 (33%)	
– Ischaemic cardiomyopathy	11 (28%)	14 (35%)	
*Heart failure medication*			0.811
– Beta blocker	37 (93%)	38 (95%)	
– ACE/ARB/ARNI	20/13/5 (95%)	25/10/2 (93%)	
– Aldosterone antagonist	32 (80%)	29 (73%)	
– SGLT2 inhibitor	5 (13%)	1 (3%)	
*QRS duration, ms*	166 ± 15	159 ± 16	0.335
*Echocardiography*			
– LV ejection fraction, %	28 ± 8	31 ± 6	0.188
– LV end-diastolic diameter, mm	60 ± 10	61 ± 9	0.791
*NYHA class*			0.738
– II	13 (32%)	15 (38%)	
– III	26 (65%)	21 (53%)	
– IV	1 (3%)	4 (10%)	

Values are *n* (%) or mean ± standard deviation

*ACE* angiotensin-converting enzyme inhibitor, *ARB* angiotensin receptor blocker, *ARNI* angiotensin receptor-neprilysin inhibitor, *BVP* biventricular pacing, *SGLT2* sodium-glucose co-transporter 2 inhibitor, *LBBP* left bundle branch pacing, *LV* left ventricular, *NYHA* New York Heart Association functional class

### Procedural outcomes of LBBP

Implantation data are summarised in Table S1 (Electronic Supplementary Material). Permanent LBBP was achieved in 31 of 40 patients (78%). Within this group of successful attempts the total procedural duration (i.e. door-to-door time) was 109 ± 32 min [median 104 min, interquartile range (IQR) 88–121 min] and the fluoroscopy time for the entire procedure was 14 ± 10 min (median 12 min, IQR 7–16 min). When patients with non-successful attempts were included in the analyses, procedural time was 122 ± 41 min [median 117 min, IQR 97–140 min] and the fluoroscopy time was 19 ± 16 min (median 15 min, IQR 7–19 min). A quarter of the patients received a dual-chamber pacemaker, whereas two-thirds of patients received a CRT‑D or dual-chamber ICD. In three patients (10%) the physician decided to implant a CRT‑P device with an RV pacing lead as a back-up for safety purposes (R-wave sensing was adequate and capture threshold low). The paced ECG patterns during LBBP were as follows: in 18 patients the paced QRS axis was inferior with leads II and III being completely positive, whereas in 13 patients the paced QRS axis was intermediate (lead II positive and lead III predominantly negative). In all patients there was discordance between aVL and aVR (see example in Fig. 1).

Satisfactory LBBP could not be achieved in nine patients. In two patients an anteroseptal myocardial infarction with local scarring on ultrasound prevented penetration of the pacing lead into the septum. In three patients the predefined electrocardiographic criteria of LBBP were not met (i.e. LVAT > 90 ms); possible reasons included latency, peripheral conduction block or increased path length in severely dilated hearts. In three cases LBBP failed because it was not possible to engage the septum owing to a lack of stable contact between the delivery sheath and the interventricular septum. In one case, lead placement was unsuccessful because the delivery sheath was not sufficiently long to reach the desired location on the septum.

Mean stimulus to peak LVAT was 81 ± 11 ms and was comparable between patients with and without ischaemic cardiomyopathy (81 ± 14 ms and 80 ± 11 ms, respectively, *p* = 0.901). Overall QRS duration decreased from 166 ± 16 ms at baseline to 123 ± 18 ms during LBBP (*p* < 0.001). In none of the patients did QRS duration increase during LBBP. Reduction in QRS duration was comparable in patients with and without ischaemic cardiomyopathy (40 ± 20 ms and 45 ± 16 ms, respectively, *p* = 0.429).

Average R‑wave amplitude and capture threshold at implantation were 11 ± 6 mV and 0.8 ± 0.4 V at 0.4 ms, respectively, and remained stable at 1 month (12 ± 5 mV and 0.8 ± 0.3 V at 0.4 ms, respectively), 3 months (13 ± 6 mV and 0.7 ± 0.3 V at 0.4 ms, respectively) and 6 months of follow-up (13 ± 6 mV and 0.7 ± 0.2 V at 0.4 ms, respectively). Pacing impedance decreased from 657 ± 128 Ω at implantation to 534 ± 87 Ω at 1 month, 520 ± 72 Ω at 3 months and 509 ± 67 Ω at 6 months.

### Complications of LBBP

In the peri- and postoperative phase no cardiac tamponade, septal coronary artery injury, interventricular fistula or pocket haematomas were seen. During follow-up visits no pocket infections, lead dislodgement or lead perforation were noticed. At every follow-up visit 12-lead ECGs were recorded, and in none of the patients was a sudden increase in LBB capture threshold > 1 V or loss of LBB capture found. In addition, none of the patients presented with stroke or transient ischaemic attack. In one patient, the RV shock lead dislodged 2 weeks after implantation, requiring lead repositioning. At the end of 6 months of follow-up there were no pocket infections and none of the pacing leads had been extracted because of lead infection/dysfunction.

### Echocardiographic and clinical outcomes of LBBP

Overall, mean LVEF improved from 28 ± 8% at baseline to 43 ± 12% at 6 months (*p* < 0.001) Data are summarised in Table S2 (Electronic Supplementary Material). The LV end-diastolic diameter decreased from 60 ± 10 mm at baseline to 54 ± 11 mm at 6 months (*p* = 0.003). Echocardiographic response (at least 5% increase in LVEF) was noticed in 25 of 29 patients (86%). Overall, NYHA functional class improved from 2.8 ± 0.5 at baseline to 1.6 ± 0.6 at 6 months (*p* < 0.001). Clinical response to LBBP (improvement by at least 1 NYHA functional class and no hospital admission for heart failure) was achieved in 24 of 29 patients (83%). The five patients who did not improve remained in the same NYHA functional class.

The four patients who did not demonstrate an echocardiographic response to LBBP also showed no improvement in NYHA functional class. However, they still had a 30- to 50-ms reduction in QRS duration with LBBP. These four patients had an ischaemic cardiomyopathy.

One patient was admitted for progressive heart failure 5 months after LBB lead placement. This patient was one of the functional (NYHA class) and echocardiographic non-responders. Two patients died during follow-up: one patient died of progressive heart failure 3 weeks after LBB lead placement; the other patient died of a pancreatic carcinoma 6 weeks after LBB lead placement, a condition of which we were unaware at the time of implantation.

### Comparison of LBBP with BVP

No significant differences in baseline characteristics (Tab. 1) were observed between the two groups. Success rates of coronary sinus lead placement (95%) and LBB lead placement (78%) showed a statistical difference (Table S1, Electronic Supplementary Material). The final position of the quadripolar coronary sinus leads was as follows: lateral in 33 patients, posterior in four patients and anterior in one patient. Total procedure time was approximately 33 min shorter with LBBP than with BVP, whereas the fluoroscopy time was comparable. At implantation, pacing parameters (i.e. R‑wave amplitude, capture threshold and impedance) were similar in the two groups. However, LBBP resulted in greater resynchronisation as compared to BVP (paced QRS duration 123 ± 18 ms vs 146 ± 26 ms, respectively, *p* < 0.001). The shortening in QRS duration as compared to their respective baseline during LBBP versus BVP was 43.8 ± 17.1 ms and 14.9 ± 25.7 ms, respectively (*p* < 0.001). Capture threshold at 6 months of follow-up was significantly higher with BVP (1.5 ± 0.6 V) than with LBBP (0.7 ± 0.2 V). Other pacing parameters, i.e. R‑wave amplitude and impedance, were not different. In addition, the percentage of LBBP or BVP was similar (both 98%).

LVEF at 6 months’ follow-up was comparable between the two groups (43 ± 12% for LBBP and 41 ± 12% for BVP, respectively, *p* = 0.720). However, there was a trend towards less improvement in LVEF with BVP when comparing the absolute increase in LVEF from their respective baseline (9.6 ± 12.1% for BVP vs 15.2 ± 11.7% for LBBP; *p* = 0.088). Similarly, there was a trend towards a smaller reduction of the LV end-diastolic diameter with BVP versus LBBP (−1.6 ± 11.8 mm vs −6.9 ± 10.3 mm, respectively, *p* = 0.090). Overall, NYHA functional class improved from 2.7 ± 0.6 at baseline to 1.6 ± 0.7 with BVP and was comparable to the improvement achieved with LBBP.

Echocardiographic response (i.e. at least 5% increase in LVEF) was achieved in 78% (28 of 36 patients) who underwent BVP and 86% (25 of 29 patients) in the LBBP group (*p* = 0.138). Similarly, clinical response (improvement by at least 1 NYHA class without a hospital admission for heart failure) was achieved in 78% (28 of 36 patients) in the BVP group and 83% (24 of 29 patients) in the LBBP group (*p* = 0.172). In the BVP group, there were two admissions for progressive heart failure. Two patients died of progressive heart failure at 1 and 5 months after the index procedure, respectively.

## Discussion

The main findings of this prospective non-randomised, single-centre study are:

1. In 78% of patients LBBP was a safe and feasible alternative approach for delivering CRT.
2. LBBP resulted in significant electrical resynchronisation and a favourable improvement in LV function and NYHA functional class.
3. Preliminary comparison between LBBP and BVP showed that the success rate of BVP was better than that of LBBP, but LBBP resulted in a greater reduction in QRS duration, whereas improvement in LVEF and LV reverse remodelling at short-term follow-up were comparable.
4. Echocardiographic and clinical response occurred equally frequently in the two groups, and hospitalisation for heart failure and all-cause mortality were similar.

### Feasibility and safety

The feasibility of using LBBP to deliver CRT was first reported by Huang et al. as a rescue pacing modality after failed coronary sinus lead placement [1]. Since then, several groups have confirmed the feasibility and safety of LBBP using the Medtronic 3830 SelectSecure pacing lead in short-term studies [1, 2, 8, 10]. In our study the implant success rate using LBBP was 78% and was comparable to data reported in recent multicentre studies evaluating the feasibility of LBBP for CRT indications [8, 11]. Our implant success rate was limited mainly by: (1) inability to engage the septum, especially in patients with septal scar tissue after myocardial infarction (*n* = 2) or severely enlarged cardiac chambers (*n* = 4); and (2) inability to meet the predefined electrocardiographic criteria for LBBP (*n* = 3). The current delivery sheath and lead were not specifically developed for LBBP and new developments in delivery sheath [e.g. increased sheath length and (steerable) sheaths offering more support] and possibly lead design (e.g. stylet driven leads) may improve the success rate. In addition, the predictive value of anteroseptal scar tissue as regards successful LBBP needs further investigation, as this could prevent futile attempts at LBBP.

LBBP can be performed swiftly, i.e. the total procedure time was approximately 30 min shorter when compared to BVP (and 20 min shorter when unsuccessful attempts were included in the analyses). Cannulation of the coronary sinus, venography and proper positioning of the coronary sinus lead can be challenging and time-consuming. In our opinion, placement of an LBB lead is a technically less complex and a more predictable procedure.

LBBP can be safely performed. No LBBP-related complications, such as lead dislodgement/perforation, a sudden increase in capture threshold or loss of LBB capture, were recorded during the 6 months of follow-up. It needs to be emphasised that the results should be interpreted with caution, since the sample size was relatively small and the follow-up period short.

With LBBP, sensing and pacing parameters were excellent when compared to BVP. At 6 months the capture threshold at 0.4 ms was 0.7 V for LBBP versus 1.5 V for BVP. Lead maturation, with subsequent low capture thresholds, may benefit from a deep intramyocardial lead position as compared to an epicardial position of coronary sinus leads.

### Clinical outcomes

LBBP resulted in a greater reduction in QRS duration when compared to BVP. With BVP, activation of the ventricles utilises non-physiological, slow cell-to-cell conduction, whereas LBBP employs the intrinsic Purkinje conduction system, leading to a narrower QRS complex. In patients with conventional bradycardia pacing indications, Sharma et al. [12] recently demonstrated that, in contrast to RV pacing, LBBP can prevent development of heart failure and mortality. RV pacing creates an activation pattern comparable to that of LBBB. Prior studies of CRT have demonstrated that QRS narrowing is associated with better clinical outcomes [13, 14].

Recently, Huang et al. [15] confirmed the feasibility and effectiveness of LBBP for CRT in preselected heart failure patients with LBBB and non-ischaemic cardiomyopathy. Implant success rates were extremely high (97%, 61 of 63 patients) with a stable capture threshold and R‑wave amplitude at 1‑year follow-up. The QRS duration narrowed from 169 ± 16 to 118 ± 12 ms during LBBP, which is comparable to our data in a mixed population with approximately 30% of patients having an ischaemic cardiomyopathy. The LVEF (33 ± 8% vs 55 ± 10%) and LV end-systolic volume (123 ± 61 ml vs 67 ± 39 ml) of their patients improved significantly compared to their baseline values. Their selection criteria—patients with typical LBBB and non-ischaemic cardiomyopathy—likely resulted in a high rate of super-responders in their study population. The largest retrospective multicentre study assessing the feasibility of LBBP for CRT was published recently by Vijayaraman et al. [8]. LBBP was achieved in 85% of the patients (277/325). However, only 39% of their patients had baseline LBBB. The outcomes in this subgroup are comparable to our results, i.e. a reduction in QRS duration from 162 ± 24 ms to 133 ± 22 ms (*p* < 0.01) with LVEF improvement from 30 ± 8% to 44 ± 11% (*p* < 0.01) at 6 months of follow-up. NYHA class improved from 2.8 ± 0.6 to 1.7 ± 0.7.

### Limitations, unknowns and future perspectives

The most important limitations of this study are its small sample size and its non-randomised design. Therefore, the results of this study must be interpreted with caution; in particular, comparisons between LBBP and BVP should be regarded as preliminary. The study was neither designed nor powered to determine differences in hard clinical endpoints. Large randomised controlled trials are needed to confirm the feasibility, long-term safety and clinical effectiveness of LBBP versus BVP for various CRT indications.

We conducted the study when LBBP was at a very early stage of clinical application, with limited criteria for LBB capture. In the current study LBB capture was confirmed by only two markers, i.e. (1) the paced QRS morphology in lead V1 demonstrating an RBB conduction delay or block pattern and (2) a stable and short LVAT. There were, however, no validated cut-off values for what the LVAT should be. In our cohort, the mean LVAT was 81 ms, which is similar to previously reported data [16–18]. However, in patients exhibiting latency or diffuse peripheral conduction disease, LVAT values may be prolonged, even with LBB capture. LVAT may also be extended in patients with severely dilated hearts, where path length to the LV lateral wall is increased. Therefore, it cannot be excluded that in three patients LBBP attempts were incorrectly considered not successful due to an LVAT > 90 ms. On the other hand, LBBP attempts in patients in whom deep LV septal pacing (with secondary activation of the left-sided Purkinje system) was performed instead of direct capture of the LBB might have been classified as successful. Whether LV septal pacing is inferior to LBBP deserves further research, especially since Mafi-Rad et al. [19] demonstrated that, in patients with sinus node dysfunction, LV septal pacing (probably with capture of distal arborisations of the left-sided Purkinje system) is able to preserve acute LV pump function (comparable to atrial pacing). In contrast, in heart failure patients the situation may be different. In the LOT-CRT study [20] patients with evidence of LBB capture had better clinical outcomes than patients with LV septal pacing. Meanwhile, several groups have been working on the establishment of novel evidence-based, rather than arbitrary, criteria for differentiation between LBBP and LV septal pacing [21–23].

Although LBBP seems a potential alternative to conventional CRT in patients with LBBB, it is still unknown which patients will benefit most from this novel technique. Heart failure patients with intraventricular conduction delay (IVCD) or RBBB might benefit less from LBBP, as LBBP may not resolve or resynchronise a delayed right ventricle. IVCD or LBBB may be the result of a diffuse, gradual conduction delay in the entire left bundle conduction system [24], which may not be synchronised by pacing at the proximal left bundle. These patients may benefit most from classic BVP. In contrast, if the LBBB can be corrected by LBBP, the region of block is most likely situated proximal to the pacing site and with intact distal Purkinje conduction [24].

Although analysis of all baseline and follow-up echocardiograms was performed in an anonymised manner, at random and after the last patient had completed follow-up, it cannot be fully excluded that outcomes may have been biased, since pacing leads in the interventricular septum were often easily visible.

In addition, delivering CRT by use of a dual-chamber pacemaker or ICD instead of a biventricular device may be helpful in limiting the continuously increasing health care costs.

## Conclusions

This study demonstrated LBBP to be a safe and feasible approach in delivering CRT in 78% of patients with favourable electrical resynchronisation and functional improvement. Results need to be interpreted with caution and need confirmation in large randomised controlled trials.

## Supplementary Information

**Table S1** Procedural data

**Table S2** Outcomes at 6 months
