# Supplementary material for: Left bundle branch pacing as an alternative to biventricular pacing for cardiac resynchronisation therapy
Source: Neth Heart J. 2022 Aug 3;31(4):140–9. doi: 10.1007/s12471-022-01712-9 (PMC10033770; doi:10.1007/s12471-022-01712-9)
Supplement: Supplementary file 1 — Table S1 Procedural data [file 12471_2022_1712_MOESM1_ESM.docx]

**Table S1** Procedural data

| **Parameter** | **LBBP** | **BVP** | ***p-value*** |
| --- | --- | --- | --- |
| Successful LBB or CS lead placement | 31 (78%) | 38 (95%) | 0.048 |
| Procedure time, min | 109±32 | 137±48 | 0.027 |
| Procedure time including unsuccessful attempts, min | 122±41 | 142±52 | 0.049 |
| Fluoroscopy time, min | 14±10 | 15±10 | 0.729 |
| Fluoroscopy time including unsuccessful attempts, min | 19±16 | 16±11 | 0.337 |
| *Implanted device* |  |  | 0.035 |
| Dual-chamber pacemaker | 8 (26%) | 0 |  |
| Dual-chamber defibrillator | 2 (6%) | 0 |  |
| CRT-P | 3 (10%) | 8 (20%) |  |
| CRT-D | 18 (58%) | 32 (80%) |  |
| *Pacing parameters* | | | |
| R-wave amplitude, mV | 11.0±6.3 | 11.9±9.2 | 0.794 |
| Capture threshold at 0.4 ms, V | 0.8±0.4 | 0.9±0.5 | 0.468 |
| Impedance, Ω | 657±128 | 598±220 | 0.169 |
| Paced QRS duration, ms | 123±18 | 146±26 | <0.001 |
| Reduction in QRS duration, ms | 43.8±17.1 | 14.9±25.7 | <0.001 |
| LVAT, ms | 81±11 | n.a. | n.a. |
| *Complications* |  |  | 0.125 |
| Pneumothorax | 0 | 2 |  |
| Pericardial effusion | 0 | 0 |  |
| Device or pocket infection | 0 | 1 |  |
| Cerebrovascular accident | 0 | 0 |  |
| RA or RV lead dislodgement | 1 | 1 |  |
| CS or LBB lead dislodgement | 0 | 1 |  |

Data based on successful implants. Values are *n* (%) or mean ± standard deviation. *p*-value indicates comparison between left bundle branch pacing (*LBBP*) and biventricular pacing (*BVP*)

*CS* coronary sinus, *CRT-P* cardiac resynchronisation therapy pacemaker, *CRT-D* cardiac resynchronisation therapy defibrillator, *LBB* left bundle branch, *LVAT* pacing stimulus to peak left ventricular activation time, *RA* right atrial, *RV* right ventricular
