# Supplementary material for: Left bundle branch pacing as an alternative to biventricular pacing for cardiac resynchronisation therapy
Source: Neth Heart J. 2022 Aug 3;31(4):140–9. doi: 10.1007/s12471-022-01712-9 (PMC10033770; doi:10.1007/s12471-022-01712-9)
Supplement: Supplementary file 2 — Table S2 Outcomes at 6 months [file 12471_2022_1712_MOESM2_ESM.docx]

**Table S2** Outcomes at 6 months

| **Parameter** | **LBBP (*n*=29)** | **BVP (*n*=36)** | ***p-value*** |
| --- | --- | --- | --- |
| *Pacing parameters* | | | |
| R-wave amplitude, mV | 12.9±5.8 | 13.4±5.9 | 0.370 |
| Capture threshold at 0.4 ms, V | 0.7±0.2 | 1.5±0.6 | 0.048 |
| Impedance, Ω | 509±67 | 608±193 | 0.417 |
| Percentage LBBP or BVP | 98±5 | 98±3 | 0.879 |
| *Echocardiographic parameters* | | | |
| LVEF, % | 43±12 | 41±12 | 0.720 |
| Increase in LVEF, % | 15.1±11.7 | 9.3±12.3 | 0.088 |
| LVEDD, mm | 54±11 | 58±11 | 0.370 |
| Reduction in LVEDD, mm | 6.9±10.3 | 1.6±11.8 | 0.090 |
| *Clinical outcomes* |  |  | 0.780 |
| Hospitalisation for heart failure | 1 | 2 |  |
| All-cause mortality | 2 | 2 |  |
| *NYHA class* |  |  | 0.611 |
| I | 13 (48) | 17 (47) |  |
| II | 12 (44) | 15 (42) |  |
| III | 2 (8) | 4 (11) |  |
| IV | 0 (0) | 0 (0) |  |

Data based on patients with successful lead implant and alive at 6 months. Values are *n* (%) or mean ± standard deviation. *p*-values indicate comparison between left bundle branch pacing (*LBBP*) and biventricular pacing (*BVP*) groups

*LVEDD* LV end-diastolic diameter, *LVEF* left ventricular ejection fraction, *NYHA* New York Heart Association
